# What are the odds you will read this article?

**DOI:** 10.3325/cmj.2019.60.53

**Published:** 2019-02

**Authors:** Pero Hrabač, Vladimir Trkulja

**Affiliations:** 1 Department of Medical Statistics, Epidemiology, and Medical Informatics, “Andrija Štampar” School of Public Health, University of Zagreb School of Medicine, Zagreb, Croatia *phrabac@hiim.hr*; 2 Department of Pharmacology, Zagreb University School of Medicine, Zagreb, Croatia

## THE CONCEPT

Biomedical research often focuses on the detection of relationships (associations) between variables (phenomena or attributes). It is important to note that association may or may not indicate a causal relationship. This depends on many factors and is beyond the scope of this text. The focus is on the odds ratio, which is one of the measures that can be used to quantify the association between two binary or dichotomous variables, ie, those that can take only two values, eg, alive/dead or ill/healthy.

## EXAMPLES

Let us assume that two variables of interest are “exposure of women to pharmacological estrogens” (yes/no) and “diagnosis of endometrial cancer” (yes/no). We may ask the question: “Is the exposure (to estrogens) associated with cancer diagnosis?” For this purpose, let us imagine two studies.

First study: At a gynecology ward, we examined 74 patients with endometrial cancer and 664 patients without cancer. We found that 56 out 74 women with cancer had previously been exposed to estrogens. The prevalence of estrogen exposure was therefore 56/74 or 0.757 or 75.7%. At the same time, among the 664 women without cancer, 274 had been exposed to estrogens, giving a prevalence of 274/664 or 0.413 or 41.3%.

Second study: 330 women are exposed to pharmacological estrogens and over the next 5 years, 56 of them are diagnosed with endometrial cancer. The cancer incidence is 56/330 or 0.170 or 17.0%. At the same time, 408 similar women are not exposed to estrogen and 18 of them are diagnosed with endometrial cancer – the incidence in this group is 18/408 or 0.044 or 4.4%.

In both studies, the results can be summarized as shown in Table 1.

**Table 1:** The results of a hypothetical study assessing the association between estrogen exposure and cancer diagnosis

	Estrogen exposure		Total
	yes	no	
Carcinoma			
yes	(a) 56	(b) 18	(a + b) 74
no	(c) 274	(d) 390	(c + d) 664
Total	(a + c) 330	(b + d) 408	738

The number of subjects in each category is the same for both studies. However, due to different study designs, conclusions that can be derived from each study are also different.

In the first study, we look at the “existing situation,” or the so-called cross-sectional convenience sample of subjects at a gynecology ward. Obviously, we cannot assess the risk of cancer based on such a study. The risk (or probability) quantifies the share of “events” in the total number of “attempts,” eg, the risk of scoring a point (“event”) in a series of 10 throws of the ball (“attempts”) in basketball. The risk is, therefore, based on incidence (ie, it is determined prospectively), whereas in the first study we can calculate only prevalence. Using the data from the first study, one can calculate the prevalence ratio and prevalence difference, as well as odds ratio. All three indicators provide a “numerical value” of an association between estrogen exposure and cancer diagnosis.

### Prevalence difference and prevalence ratio

In women with cancer, the prevalence of estrogen exposure was 75.7% (see above). In women without cancer, the prevalence was 41.3%. If one is to “look at the table” from a “different angle,” then the prevalence of cancer among women exposed to estrogen is 56/330 or 17% and among nonexposed women it is 18/408 or 4.4%.

Prevalence difference is therefore 75.7-41.3 = 34.4%, or alternatively, 17.0-4.4 = 12.6%

Prevalence ratio is 75.7/41.3 = 1.83, or alternatively, 17.0/4.4 = 3.85

Interpretation: The proportion of women with a history of estrogen exposure among women with cancer is absolutely 34.4% higher and relatively 83% higher than among women without cancer. Alternatively, the proportion of women with cancer among women exposed to estrogens is absolutely 12.6% and relatively 3.85 times higher than among women not exposed to estrogens.

Conclusion: Having endometrial cancer is related to previous exposure to estrogens.

**Note**: Prevalence difference or ratio differs depending on the way we “look at the table,” but in both cases both indices suggest an association between estrogen exposure and cancer.

### Odds ratio

Let us first define the term and explain how it differs from probability (risk). As mentioned, probability is a proportion of events in a number of attempts. For example, the probability of getting “six” in one throw of a fair dice is 1/6 = 16.7%. Probability can have values between 0 and 1 (ie, 0% and 100%). The probability of an unavoidable event (the sun rising tomorrow morning) is 100%. The probability of an impossible event (getting seven in a single throw of dice) is 0%. The odds of an event are the probability that the event will occur divided by the probability that it will not occur. Therefore, odds = (probability of event) / (1 – probability of event). For example, the odds of getting six in one roll of dice are 1/6 (probability of getting six) divided by 5/6 (probability of not getting six). Therefore, the odds of getting a six are [1/6]/[5/6] = 1/5 = 0.20. The odds of an event can range from 0 to infinity. Odds lower than 1 mean that it is more likely that the event will not occur than that it will occur. Odds above 1 mean that it is more likely that the event will occur. Odds ratio quantifies the relationship between two binary variables based on a comparison of the odds of the event under two different conditions. In the case discussed here, the event is cancer diagnosis, and two conditions are the presence or absence of estrogen exposure. The odds ratio of interest is the ratio of the odds of estrogen exposure in women with cancer and the odds of estrogen exposure in women without cancer. Therefore:

Odds of estrogen exposure in women with cancer: 56/18 = 3.11.

Odds of estrogen exposure in women without cancer: 274/390 = 0.70.

Odds ratio is therefore: 3.11/0.70 = 4.42.

Interpretation: The odds of finding previous estrogen exposure are 4.42 times higher among women with endometrial cancer than among women without cancer.

Conclusion: The presence of endometrial cancer is associated with a previous exposure to pharmacological estrogens.

**Note**: If one is to “look at the data” another way around, ie, at the odds of finding cancer in exposed women and the odds of finding cancer in non-exposed women, there would be the following situation:

Odds of cancer in exposed women: 56/274 = 0.204

Odds of cancer in non-exposed women: 18/390 = 0.046

Odds ratio: 0.204/0.046 = 4.42

Hence, odds ratio gives an identical numerical “value” of an association, irrespective of the way of “looking” at the data.

However, there are two questions still remaining. First, the one from the title of this paper – what are the odds that a person in the possession of this issue of the *Croatian Medical Journal* will read this article? And second, what about the study following women taking estrogen for 5 years? Read more about these topics in our next column!

